# Fully-connected network-based prediction model for lymph node metastasis in clinical early-stage endometrial cancer: development and validation in two centers

**DOI:** 10.3389/fonc.2025.1627662

**Published:** 2025-08-25

**Authors:** Shuyan Cai, Yuzhen Huang, Wei Liu, Yulan Ren, Huaying Wang, Zhiying Xu, Yu Xue, Yiqin Wang, Xiaojun Chen

**Affiliations:** ^1^ Department of Gynecology, Obstetrics and Gynecology Hospital of Fudan University, Shanghai, China; ^2^ Shanghai Key Laboratory of Female Reproductive Endocrine-Related Diseases, Shanghai, China; ^3^ Department of Gynecologic Oncology, Fudan University Shanghai Cancer Center, Shanghai, China; ^4^ Department of Pathology, Obstetrics and Gynecology Hospital of Fudan University, Shanghai, China; ^5^ Department of Obstetrics and Gynecology, Tenth People’s Hospital of Tongji University, Shanghai, China

**Keywords:** early-stage, endometrial cancer, lymph node metastasis, fully-connected network, prediction model

## Abstract

**Objective:**

The risk of lymph node metastasis significantly influences the choice of surgical strategy for patients with early-stage endometrial cancer. While sentinel lymph node dissection can be considered in clinically early-stage endometrial cancer, lymph node evaluation might be omitted in patients with very low risk of lymph node metastasis. This study aims to develop a predicting model for lymph node metastasis in these patients, identifying potential metastases as thoroughly as possible to provide clinicians with a preoperative reference that helps in decisions about surgical procedures and treatments.

**Materials and Methods:**

We retrospectively collected data from 4,400 cases across two centers to develop a predictive model for lymph node metastasis in patients with early-stage endometrial cancer using a Fully-connected (FC) Network. Internal validation was performed, and an additional 750 cases were prospectively collected from subcenter 1 for external validation. After comparing commonly used imputation methods, missing values were filled using the K-Nearest Neighbors (KNN) for the highest sensitivity of the model. The model was evaluated by precision, sensitivity, specificity, and overall accuracy. The performance of the model was compared to other machine-learning models. The risk stratification was divided by 1%, 5%, and 25%. Combining the results of Logistic regression, the pathological subtype-specific nomograms were constructed and served as alternatives to the FC Network.

**Results:**

The FC Network achieved the highest sensitivity—0.982 in internal validation and 0.900 in external validation—demonstrating exceptional performance in identifying patients with probable lymph node metastasis compared to other machine-learning methods. Considering the prognostic implications of histological subtypes, subtype-specific nomograms were constructed, achieving AUCs of 0.810/0.784/0.834 for non-aggressive and 0.726/0.810/0.650 for aggressive subtypes across the training, internal, and external cohorts.

**Conclusions:**

The model proposed in this study can be used for risk prediction of lymph node metastasis in early-stage patients. The nomograms can be used as a feasible and easily used alternative for the model.

## 1 Introduction

Endometrial cancer (EC) is the fourth most common female malignant tumor (1). According to the data released by GLOBOCAN2022, the incidence of endometrial cancer has been rising in many countries, posing a serious threat to women’s health (2). The early symptoms of endometrial cancer include abnormal uterine bleeding, especially in postmenopausal women, which could be detected without specific examination (3, 4). In recent years, improved health awareness has led to earlier diagnoses, increasing the possibility of complete recovery. Lesions confined to the uterus without evidence of extrauterine metastasis confirmed by preoperative imaging evaluation were considered clinically early-stage disease. Patients at this stage have less than a 10% chance of lymph node metastasis. EC encompasses a range of pathological subtypes, including serous and clear cell carcinomas, which are associated with higher risks of lymph node metastasis. Current guidelines recommend total hysterectomy with bilateral salpingo-oophorectomy (TH/BSO) and surgical staging for patients suitable for primary surgery, with lymph node assessment being a key component. Lymph node dissection can assess lymph node involvement, it does not improve the prognosis for these patients (5, 6). Conversely, excessive lymph node dissection may cause postoperative complications such as lower limb lymphedema, which is strongly related to the decreased quality of life (7), and did not improve disease-free or overall survival (8). However, there are still some patients with clinical early-stage endometrial cancer who have lymph node metastases at the time of diagnosis (9, 10). If these patients had not received appropriate treatments, they would have a high risk of postoperative recurrence. Therefore, it is crucial to find a non-surgical way to identify those with a relatively high risk of lymph node metastasis in patients with clinical early-stage endometrial cancer, especially before surgery.

A study extracted features from magnetic resonance imaging (MRI) to construct a nomogram for prediction of risk of lymph node metastasis in clinically early-stage endometrial cancer patients, demonstrating good performance (11). Another study combined CA125 and HE4 to predict the risk of lymph node metastasis in early-stage patients, finding that HE4 alone could achieve good sensitivity. In our study, we aimed to establish a predictive model for lymph node metastasis in patients with clinically early-stage endometrial cancer using a Fully-connected network—a method widely used in other medical research. We comprehensively utilized general information, classical pathological parameters, laboratory tests, imaging data, and molecular classification. Incorporating various types of data allows for a more accurate preoperative evaluation of patients, while employing a more efficient and accurate machine-learning method- Fully-connected Network enhances the model’s predictive effectiveness.

## 2 Materials and methods

This study was conducted according to the Declaration of Helsinki and approved by the Ethical Committee of Obstetrics and Gynecology Hospital of Fudan University (2020-169) and the Ethical Committee of Cancer Hospital of Fudan University.

### 2.1 Study cohorts and the subgroups

4400 cases of endometrial cancer who received primary surgery in Obstetrics and Gynecology Hospital of Fudan University (defined as Subcenter 1) between January 2016 and February 2023 were enrolled in this study. And 1995 cases who received primary surgery between January 2013 and October 2020 in Cancer Hospital of Fudan University (defined as Subcenter 2) were collected. The variables included in this model collected by these two centers during these time frames were relatively uniform and complete. The inclusion criteria for the study were: (1) pathological diagnosis of EC by preoperative endometrial biopsy; (2) preoperative imaging (chest CT and pelvic/abdomen enhanced CT/MRI) suggesting tumor confinement to the uterine body, without evidence of extrauterine involvement; (3) comprehensive staging surgery performed, including total hysterectomy + bilateral salpingectomy +/- bilateral oophorectomy + pelvic lymphadenectomy or sentinel lymph node biopsy +/- para-aortic lymphadenectomy or biopsy +/- omentectomy.

**Figure 1:**
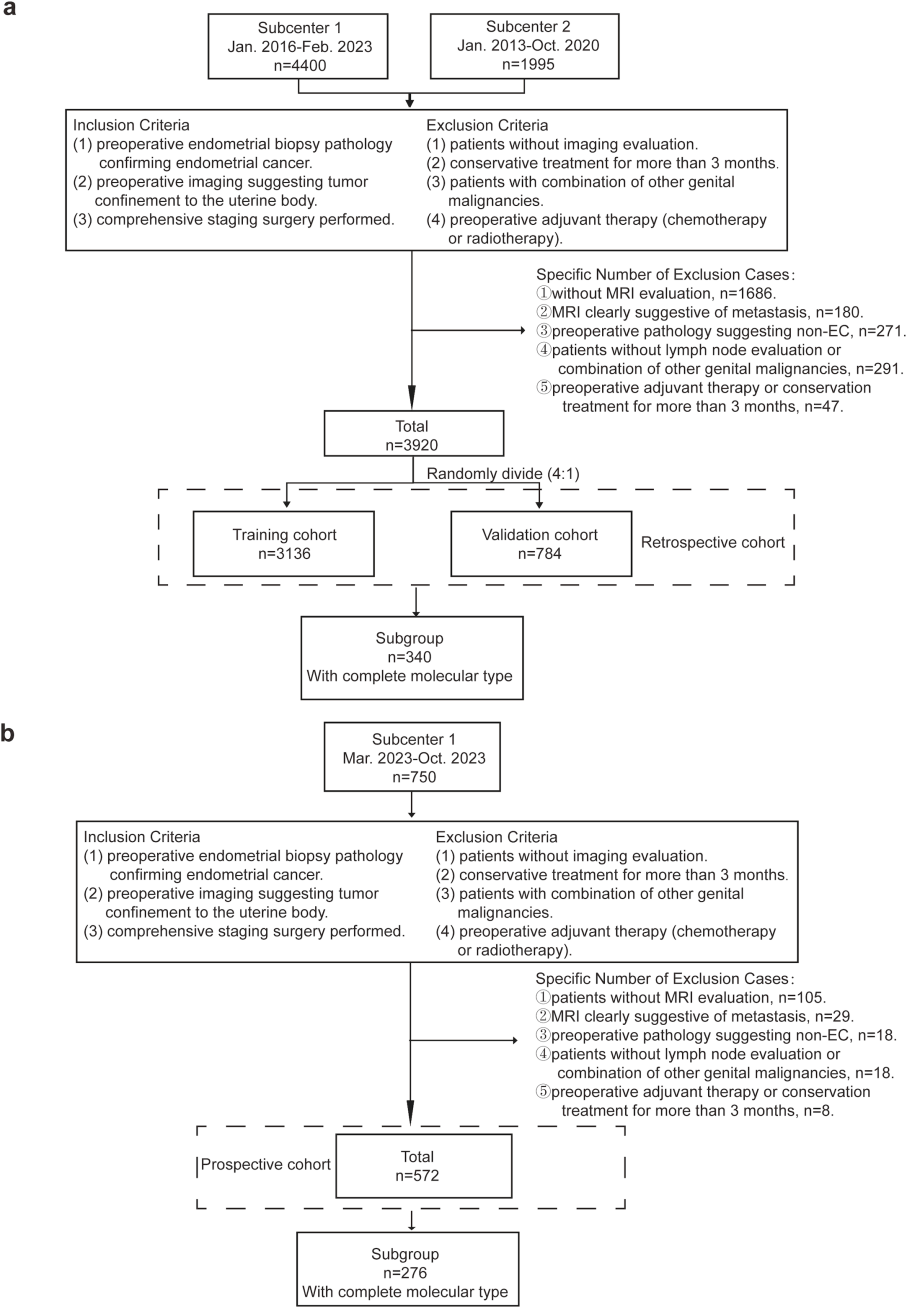
Inclusion and Exclusion Criteria of the Cohorts. **(a)** 4400 cases were retrospectively collected from Subcenter 1 (the Obstetrics and Gynecology Hospital of Fudan University) and Subcenter 2 (the Cancer Hospital of Fudan University). The final number of included cases was 3920. Cases were randomly divided into the training cohort and internal validation cohort. 340 cases of those with complete molecular type data were extracted for the subgroup. **(b)** 750 cases from subcenter 1 (the Obstetrics and Gynecology Hospital of Fudan University) were prospectively collected for external validation. The final number of included cases was 572. 276 cases of those with complete molecular type data were extracted for the subgroup.

The exclusion criteria for the study were: (1) patients without preoperative imaging evaluation; (2) patients with conservative treatment for more than 3 months; (3) had other reproductive system malignancies; (4) received preoperative adjuvant chemotherapy or radiotherapy. The detailed number of cases is shown in **Figure 1**. A final number of 3920 cases were retrospectively included in this study. The retrospective cohort was randomly divided into the training and internal validation cohorts in a ratio of 4:1. Following the development of the model, we prospectively collected an additional 750 cases from Subcenter 1 between March 2023 and October 2023 for the purpose of external validation. Based on the same inclusion criteria, a total of 572 patients were ultimately included in the external validation cohort. To evaluate the impact of molecular classification on model performance, a predefined subgroup was established, consisting of cases with complete molecular subtype information—340 from the retrospective cohort and 276 from the prospective cohort.

### 2.2 Data collection

The following data related to the risk of lymph node metastasis were collected for the subsequent analysis.

General information: age, height, weight, BMI, menopause, hypertension, diabetes.

Laboratory tests: estradiol (E2, pmol/L); progesterone (P, nmol/L); testosterone (T, nmol/L); follicle stimulating hormone (FSH, mIU/m); luteinizing hormone (LH, mIU/m); sex hormone-binding globulin (SHBG, nmol/L); triglyceride (TG, mmol/L); total cholesterol (TC, mmol/L); high-density lipoprotein (HDL, mmol/L); low-density lipoprotein (LDL, mmol/L); apolipoprotein A (APOA, g/L); apolipoprotein B (APOB, g/L); fasting plasma glucose (FPG, mmol/L); glycated hemoglobin AIC (HBA1C, %); ALP, (U/L); CA125, (U/mL); HE4, (pmol/L).

Preoperative ultrasound: thickness of the endometrium (single layers, mm); size of the lesion (longest diameter<2cm, ≥2cm); lesion-myometrial interface (clear, unclear, myometrial invasion).

Preoperative pelvic MRI: the size of the lesion (<2cm, ≥2cm); depth of myometrial invasion (no, superficial, deep); cervical involvement (with or without); enlarged pelvic lymph node but no definitive sign of metastasis (with or without); enlarged para-aortic lymph node but no definitive sign of metastasis (with or without).

Preoperative CT of the upper abdomen: enlarged retroperitoneal lymph node but no definitive sign of metastasis (with or without).

Usually, the short axis of more than 8mm in pelvic lymph nodes and 10mm in abdominal lymph nodes is considered enlarged and has possible metastasis. Regardless of size, lymph nodes with irregular boundaries or similar signal intensity to the primary tumor are considered to have metastasis (12).

Pathological results: preoperative pathological type (endometrioid, serous, mixed, clear cell, high-grade adenocarcinoma) (13).

Immunohistochemistry: MMR (dMMR, pMMR); p53 (wild type, mutant type); PTEN (negative, positive); ER (≤1%, 1-10%, >10%); PR (≤1%, 1-10%, >10%); Ki67 (percentage of positive cells).

Molecular classification: POLEmut, dMMR, NSMP, P53abn (14).

The outcome event—presence or absence of lymph node metastasis—was confirmed by postoperative pathological reports.

### 2.3 Establishment and evaluation of the model

#### 2.3.1 Establishment of the model based on fully-connected network

We could not determine which specific variables plays the most significant role in predicting lymph node metastasis in FC Network at the beginning of our study. Therefore, we included as many variables as possible to provide a more accurate representation of the patient’s condition. A total of 41 preoperatively obtainable variables were included in the model construction process. Continuous variables were directly used as inputs to the model, while categorical variables were one-hot encoded and transformed into distinct vectors. We implemented a Fully Connected Neural Network (also known as a Feedforward Neural Network or Multi-Layer Perceptron, MLP) using the PyTorch framework (15). This network, well-suited for binary classification problems (16), comprised one input layer, four hidden layers, a dropout layer, and one final output layer. The hidden layers contained 256, 128, 64, and 20 neurons, respectively. A dropout layer with a 50% rate was introduced between layers to prevent overfitting, supported by a weight decay coefficient of 0.0001 to further constrain model complexity.

The output layer generated a two-dimensional probability distribution via a SoftMax activation function, which enabled binary classification. The network was trained end-to-end using the Adam optimization algorithm with a learning rate of 0.0001. Cross-entropy was used as the loss function, and training was performed on an NVIDIA GTX 2080Ti GPU. Throughout training, backpropagation was applied to iteratively update model parameters until convergence was achieved. The structure and data flow of the network are illustrated in **Supplementary Figure 1**. To interpret variable contributions, we applied three complementary strategies: (1) calculating the gradient of model output with respect to each input, scaled by the standard deviation of feature distribution; (2) aggregating gradients by factor when variables spanned multiple encoded features; and (3) applying mean imputation to simulate feature exclusion and assessing its effect on model output.

#### 2.3.2 Evaluation of the model

The model was evaluated by precision, sensitivity, specificity, and overall accuracy. The internal and external validation cohorts were used to test the model’s performance. Based on the actual clinical application, lymph node metastasis was hard to discover, and the severe consequence of missed discovery of lymph node metastasis would threaten patients’ prognoses. Thus, we recognized sensitivity as the primary metric we focused on in this study to screen out patients with lymph node metastases as much as possible. According to a large cohort study, we considered the probability of less than 1% as extremely low risk, 5% as low risk, 5% to 25% as medium risk, and more than 25% as high risk based on another study (17). Therefore, the model will consider “metastatic” with a predicted probability greater than 1%.

### 2.4 Comparison of different missing data fill-in methods

Due to the long period of data collection and the emergence of newly developed tests, some of the data needed to be included in some cases. Thus, we used five data fill-in methods (mean, median, plurality, fixed constant, and K-nearest neighbor) to fill in the missing values and then constructed the models based on the cohorts filled in with these five methods. The method with the highest sensitivity was preserved and applied to the internal and external validation cohorts. Subsequently, five models were constructed based on these different cohorts. The performance of the models was recorded in **Supplementary Table 1**. The model with missing values filled by the Constant has the highest overall accuracy (0.186), precision (0.078), and specificity (0.126), and the model filled in by Median has the highest AUC value (0.773). In terms of sensitivity, the model filled in by Mean, Constant, and KNN achieved the highest, 0.982, followed by Median (0.964) and Most Frequent (0.864). The Receiver Operating Characteristic (ROC) curves were recorded in **Supplementary Figure 2**. Since sensitivity reflects the ability to correctly identify patients with lymph node metastasis—which is crucial for their prognosis. Three methods can achieve the best sensitivity, proving these three ways’ feasibility. Considering the characteristics of the values in our cohorts, KNN would be the most suitable method to fill in the missing values. Therefore, we applied KNN, which also proved to be effective in other studies (18–21), to fill in the missing values in three cohorts to optimize the predictive effectiveness of the FC Network in the training cohort for the subsequent analyses.

### 2.5 Comparison of the model with other machine-learning methods

We also constructed other machine-learning models based on the same training cohort after handling the missing data to compare the effectiveness of these models. These selected machine-learning methods were Entropy Decision Tree, Regression Decision Tree, Gaussian Plain Bayes, Polynomial Plain Bayes, Bernoulli Plain Bayes, K Nearest Neighbors, Logistic Regression, Support Vector Machines, and Random Forest.

Entropy Decision Tree: This is a decision tree algorithm designed for classification problems. It selects the optimal feature for data splitting based on information entropy, creating a tree-like structure where each leaf node represents a class. Regression Decision Tree: This decision tree algorithm is used for regression tasks. It fits data by selecting the best features and thresholds, resulting in a tree-like structure that predicts numerical values. Gaussian Naive Bayes (Gaussian NB): A classification algorithm based on Bayes’ theorem. It assumes that the features follow a Gaussian (normal) distribution and is well-suited for handling continuous feature data. Multinomial Naive Bayes (Multinomial NB): A naive Bayes algorithm typically used for text classification. It assumes that features are discrete, often represented as integer counts. Bernoulli Naive Bayes (Bernoulli NB): Another naive Bayes algorithm, typically applied to binary features (0 and 1). It is often used for classification tasks involving binary representations of text. K-Nearest Neighbors (K Neighbors): A supervised learning algorithm used for both classification and regression tasks. It classifies new instances or predicts values based on the distance metric of the K nearest neighbors. Logistic Regression: A linear model used for binary and multiclass classification problems. It applies a sigmoid function to map the linear combination of features to probability values for classification. Support Vector Machine (SVM): A supervised learning algorithm that finds the optimal hyperplane to separate data, maximizing the margin between classes. Random Forest: An ensemble learning method based on multiple decision trees. It is used for classification and regression tasks, reducing overfitting and improving performance by randomly sampling data and features.

These machine-learning methods have been applied to medical research widely (22–25). As mentioned above, the sensitivity was the main index we compared.

### 2.6 Construction of the simplified nomograms

To facilitate practical use and offer an alternative to the model in certain situations, we developed simplified nomograms by combining the results of the Top 10 variables ranked by weights in FC Network with the results of related variables in Logistic Regression analysis based on different pathological subtypes. Initially, we performed single factor and multi-factor Logistic Regression analyses. Variables that remained statistically significant and overlapped with the top 10 weighted variables from the FC Network were selected for constructing the nomograms which were also the commonly-used variables in clinical applications.

### 2.7 Statistical analysis

Differences between cohorts were assessed using the following statistical tests: For continuous variables with a normal distribution, a t-test was applied; for those not fitting a normal distribution, the Mann-Whitney U test was used. Categorical variables were analyzed with the chi-square test. The comparison between FC Network with other machine-learning methods were mainly focus the term of sensitivity. The DeLong test compared the AUC values of the models. For the construction of nomogram, Logistic regression was used. Univariate and multivariate regression analyses were conducted using SPSS Statistics 29. Nomogram visualization was performed with R software (version 4.1.0). Statistical significance was defined as a two-tailed P-value less than 0.05.

## 3 Results

### 3.1 The clinical baseline of three cohorts

**Table 1** presents the clinical characteristics and the number of missing values in the training, internal validation, and external validation cohorts. Baseline characteristics showed no significant differences between the training and internal validation cohorts, indicating consistency between these datasets. Ultimately, we included 3,920 patients in the training and internal validation cohorts and an additional 572 cases in the external validation cohort. The 3,920 patients were randomly divided into training and internal validation groups at a 4:1 ratio. In the training cohort, 218 patients (6.95%) had lymph node metastases, while 55 patients (7.02%) in the internal validation cohort had metastases. In the external validation cohort, there were 30 metastatic cases (5.24%). The proportion of missing data, which is due to the high cost and extended data collection, was recorded in **Table 1**.

**Table 1:** Clinical characteristics and no. of missing data of training and internal/external validation cohorts.

Variable	Training cohort (*n*=3136)	Internal validation cohort (*n*=784)	*P* value	External validation cohort (*n*=572)
General Information				
Age	54.00 (49.00-60.00)	54.00 (49.00-60.00)	0.748	54.00 (48.00-59.00)
Height (m)	1.60 (1.56-1.63)	1.60 (1.57-1.63)	0.352	1.60 (1.57-1.63)
No. of missing data	44 (1.40%)	9 (1.15%)		0
Weight (kg)	61.00 (55.00-69.00)	62.00 (55.88-68.60)	0.720	62.00 (55.58-70.00)
No. of missing data	4(0.13%)	0		0
BMI (kg/m^2^)	24.24 (22.03-27.05)	24.24 (22.17-26.62)	0.991	24.54 (22.16-27.30)
No. of missing data	44 (1.40%)	9 (1.15%)		0
Menopause			0.414	
No	864 (27.55%)	204 (26.02%)		236 (41.26%)
Yes	2272 (72.45%)	580 (73.98%)		336 (58.74%)
Hypertension			0.247	
No	2175 (69.36%)	561 (71.56%)		417 (72.90%)
Yes	961 (30.64%)	223 (28.44%)		155 (27.10%)
Diabetes			0.957	
No	2688 (85.71%)	671 (85.59%)		476 (83.22%)
Yes	447 (14.29%)	113 (14.41%)		96 (16.78%)
Hormone				
E2 (pmol/L)	102.76 (51.38-238.55)	99.09 (55.05-215.00)	0.593	68.00 (37.00-209.00)
No. of missing data	2425 (77.33%)	635 (80.99%)		317 (55.42%)
P (nmol/L)	1.21 (0.70-1.96)	1.14 (0.67-1.87)	0.610	0.76 (0.33-1.36)
No. of missing data	2458 (78.38%)	638 (81.38%)		321 (56.12%)
T (nmol/L)	1.25 (0.83-1.77)	1.37 (0.82-1.90)	0.216	1.05 (0.56-1.53)
No. of missing data	2461 (78.48%)	637 (81.25%)		317 (55.42%)
FSH (mIU/m)	36.96 (7.75-70.85)	42.19 (7.77-72.70)	0.802	31.98 (6.01-65.43)
No. of missing data	2428 (77.42%)	634 (80.87%)		317 (55.42%)
LH (mIU/m)	16.54 (5.83-29.52)	19.54 (8.21-29.06)	0.152	14.64 (4.88-28.72)
No. of missing data	2441 (77.84%)	634 (80.87%)		316 (55.24%)
SHBG (nmol/L)	44.20 (30.45-65.90)	44.00 (28.83-63.85)	0.570	41.80 (28.50-68.00)
No. of missing data	2621 (83.58%)	678 (86.48%)		403 (70.45%)
Blood lipids				
TG (mmol/L)	1.52 (1.07-2.18)	1.56 (1.09-2.19)	0.358	1.33 (0.94-1.96)
No. of missing data	1041 (33.20%)	272 (34.69%)		36 (6.29%)
TC (mmol/L)	5.15 (4.51-5.82)	5.13 (4.51-5.81)	0.970	4.79 (4.12-5.49)
No. of missing data	1042 (33.23%)	273 (34.82%)		36 (6.29%)
HDL (mmol/L)	1.27 (1.09-1.47)	1.24 (1.06-1.46)	0.275	1.26 (1.06-1.49)
No. of missing data	1050 (33.48%)	274 (34.95%)		43 (7.52%)
LDL (mmol/L)	3.28 (2.77-3.84)	3.22 (2.83-3.88)	0.816	3.05 (2.47-3.61)
No. of missing data	1050 (33.48%)	273 (34.82%)		38(6.64%)
APOA (g/L)	1.38 (1.14-1.57)	1.36 (1.13-1.57)	0.505	1.36 (1.25-1.49)
No. of missing data	1256 (40.05%)	339 (43.24%)		51 (8.92%)
APOB (g/L)	0.99 (0.85-1.15)	0.99 (0.87-1.17)	0.330	0.92 (0.78-1.05)
No. of missing data	1255 (40.02%)	340 (43.37%)		51 (8.92%)
Glucometabolic				
FPG (mmol/L)	5.40 (5.00-5.90)	5.40 (5.00-6.00)	0.761	5.20 (4.80-5.60)
No. of missing data	1127 (35.94%)	283 (36.10%)		36 (6.29%)
HBA1C (%)	5.70 (5.40-6.10)	5.70 (5.40-6.10)	0.759	5.70 (5.40-6.10)
No. of missing data	2156 (68.75%)	562 (71.68%)		89 (15.56%)
ALP (U/L)	78.00 (63.52-95.38)	77.00 (63.00-94.00)	0.356	77.00 (63.25-90.00)
No. of missing data	42 (1.34%)	13 (1.66%)		10 (1.75%)
Tumor marker				
CA125 (U/mL)	17.80 (12.46-27.19)	17.46 (12.58-26.47)	0.814	16.20 (11.43-25.40)
No. of missing data	206 (6.57%)	50 (6.38%)		14 (2.45%)
HE4 (pmol/L)	60.05 (48.48-81.90)	59.30 (49.40-79.38)	0.853	59.30 (48.10-79.50)
No. of missing data	389 (12.40%)	100 (12.76%)		23 (4.02%)
Preoperative ultrasound				
Thickness of Endometrium(mm)	6.00 (2.00-10.00)	6.00 (2.00-11.00)	0.555	5.00 (2.00-10.00)
No. of missing data	1381 (44.04%)	361 (46.05%)		105 (18.36%)
Size of Lesion			0.463	
<2cm	447 (14.25%)	118 (15.05%)		418 (73.08%)
≥2cm	647 (20.63%)	153 (19.52%)		132 (23.08%)
No. of missing data	2042 (65.11%)	513 (65.43%)		22 (3.85%)
Lesion-myometrial interface			0.637	
Clear	1702 (54.27%)	410 (52.30%)		427 (74.65%)
Unclear	460 (14.67%)	111 (14.16%)		113 (19.76%)
Myometrial Invasion	54 (1.72%)	17 (2.17%)		6 (1.05%)
No. of missing data	920 (29.34%)	246 (31.38%)		26 (4.55%)
Preoperative pelvic MRI				
Size of Lesion			0.358	
<2cm	1086 (34.63%)	255 (32.53%)		311 (54.37%)
≥2cm	1110 (35.40%)	286 (36.48%)		261 (45.63%)
No. of missing data	940 (29.97%)	243 (30.99%)		0
Myometrial Invasion			0.336	
No	1533 (48.88%)	362 (46.17%)		227(39.69%)
Shallow	1275 (40.66%)	341 (43.49%)		254 (44.41%)
Deep	327 (10.43%)	81 (10.33%)		91 (15.91%)
No. of missing data	1 (0.03%)	0		0
Cervical Involvement			0.457	
Yes	142 (4.53%)	41 (5.23%)		32 (5.59%)
No	2993 (95.44%)	742 (94.64%)		540 (94.41%)
No. of missing data	1 (0.03%)	1 (0.13%)		0
Enlarged Pelvic Lymph Node			0.127	
No	3036 (96.81%)	749 (95.54%)		538 (94.06%)
Yes	99 (3.16%)	34 (4.34%)		34 (5.94%)
No. of missing data	1 (0.03%)	1 (0.13%)		0
Enlarged Para-aortic Lymph Node			0.885	
No	3129 (99.78%)	783 (99.87%)		572 (100.00%)
Yes	3 (0.10%)	0		0
No. of missing data	4 (0.13%)	1 (0.13%)		0
Preoperative CT of Upper Abdomen				
Enlarged Retroperitoneal Lymph Node			0.870	
No	2144 (68.37%)	523 (66.71%)		414 (72.38%)
Yes	45 (1.43%)	11 (1.40%)		17 (2.97%)
No. of missing data	947 (30.20%)	250 (31.89%)		141 (24.65%)
Preoperative Pathological Type			0.833	
Endometrioid	2821 (89.96%)	701 (89.41%)		497 (86.89%)
Serous	57 (1.82%)	17 (2.17%)		9 (1.57%)
Mixed	27 (0.86%)	9 (1.15%)		4 (0.70%)
Clear Cell	39 (1.24%)	13 (1.66%)		9 (1.57%)
High Grade Adenocarcinoma	151 (4.82%)	34 (4.34%)		35 (6.12%)
Others	41 (1.31%)	10 (1.28%)		18 (3.15%)
Immunohistochemistry				
MMR			0.426	
dMMR	690 (22.00%)	183 (23.34%)		144 (25.17%)
pMMR	1937 (61.77%)	473 (60.33%)		392 (68.53%)
No. of missing data	509 (16.23%)	128 (16.33%)		36 (6.29%)
p53			0.981	
Wild Type	2406 (76.72%)	603 (76.91%)		459 (80.24%)
Mutant Type	333 (10.62%)	83 (10.59%)		81 (14.16%)
No. of missing data	397 (12.66%)	98 (12.50%)		32 (5.59%)
PTEN			0.956	
Negative	1535 (48.95%)	378 (48.21%)		368 (64.34%)
Positive	812 (25.89%)	202 (25.77%)		165 (28.85%)
No. of missing data	789 (25.16%)	204 (26.02%)		39 (6.82%)
ER			0.625	
≤1%	181 (5.77%)	51 (6.51%)		38 (6.64%)
1-10%	370 (11.80%)	86 (10.97%)		17 (2.97%)
>10%	2172 (69.26%)	542 (69.13%)		484 (84.62%)
No. of missing data	413 (13.17%)	105 (13.39%)		33 (5.77%)
PR			0.721	
≤1%	401 (12.79%)	104 (13.27%)		62 (10.84%)
1-10%	620 (19.77%)	163 (20.79%)		38 (6.64%)
>10%	1703 (54.30%)	415 (52.93%)		439 (76.75%)
No. of missing data	412 (13.14%)	102 (13.01%)		33 (5.77%)
Ki67	40% (20-60%)	40% (20-60%)	0.939	30% (20-50%)
No. of missing data	419 (13.36%)	104 (13.27%)		35 (6.12%)
Molecular Type			0.326	
POLE*mut*	34 (1.08%)	5 (0.64%)		43 (7.52%)
dMMR	90 (2.87%)	24 (3.06%)		77 (13.46%)
NSMP	101 (3.22%)	16 (2.04%)		115 (20.10%)
P53abn	61 (1.95%)	9 (1.15%)		41 (7.17%)
No. of missing data	2850 (90.88%)	730 (93.11%)		296 (51.75%)
Lymph Node Metastasis			0.987	
No	2918 (93.05%)	729 (92.98%)		542 (94.76%)
Yes	218 (6.95%)	55 (7.02%)		30 (5.24%)

P value showed the statistical analysis between the training cohort and validation cohort. For variables conforming to normal distribution, a t-test was used. For continuous variables that did not conform to normal distribution, the Mann-Whitney test was used. For categorical variables, the chi-square test was used. P<0.05 was considered statistically different.

### 3.2 The outstanding performance of the model based on FC network

**Figure 2:**
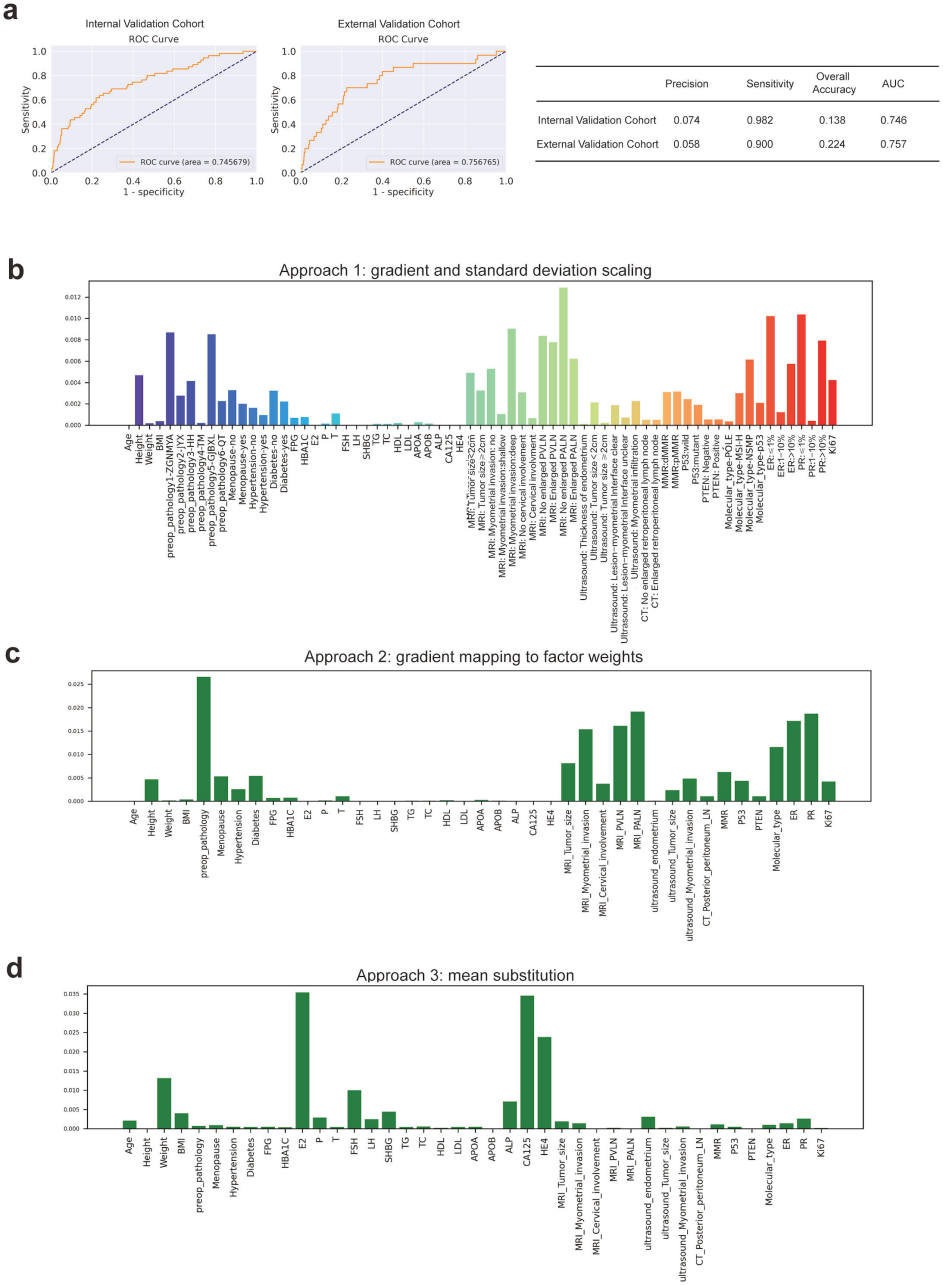
The Construction and Estimation of FC Network. **(a)** ROC curves in internal validation and prospective external validation cohorts. **(b)** Approach 1: gradient and standard deviation scaling. **(c)** Approach 2: gradient mapping to factor weights. **(d)** Approach 3: mean substitution.

Based on the same training cohort filled in the missing value by KNN, we then constructed other machine-learning models to compare the performances of these models. The FC Network showed the highest sensitivity which demonstrated outstanding performance in screening out probable patients with lymph node metastasis. The chosen traditional machine-learning methods were Decision Tree Entropy, Decision Tree Regressor, Gaussian NB, Multinomial NB, Bernoulli NB, K Neighbors, Logistic Regression, SVM, and Random Forest. The results are recorded in **Table 2**. Random forest has the highest overall accuracy, which is 0.931. Also, the random forest has the highest precision and specificity; both were 1. However, the FC Network has the best sensitivity (0.982), followed by Gaussian NB (0.618) and Multinomial NB (0.618), which indicates outstanding performance in screening out patients with probable lymph node metastasis beyond these other machine-learning methods. We further used the external validation cohort to verify the performance of the FC Network, and it reached a sensitivity of 0.900. Meanwhile, the AUC values of the FC Network were 0.746 in the internal validation cohort and 0.757 in the external validation cohort, respectively. The results are shown in **Figure 2a**.

**Table 2:** Comparison between fully-connected network and other machine-learning methods.

Method	Sensitivity	Specificity	Precision	Overall accuracy
FC Network	0.982	0.074	0.074	0.138
Decision Tree Entropy	0.182	0.933	0.170	0.880
Decision Tree Regressor	0.200	0.940	0.200	0.888
Gaussian NB	0.618	0.778	0.174	0.767
Multinomial NB	0.618	0.717	0.142	0.710
Bernoulli NB	0.400	0.925	0.286	0.888
K Neighbors	0.055	0.995	0.429	0.929
Logistic Regression	0.073	0.993	0.444	0.929
SVM	0.000	0.999	0.000	0.929
Random Forest	0.018	1.000	1.000	0.931

### 3.3 The influence of molecular classification on the model

The addition of molecular classification may enhance the predictive power of the model after comparing the models with or without molecular classification. As previously mentioned, the proportion of missing data for molecular classification was high in both the retrospective and prospective cohorts. Nevertheless, given the growing consensus on its importance, we included molecular classification as one of the variables to broaden the model’s applicability in future studies. Therefore, we extracted cases with complete molecular classification (340 cases in the retrospective cohort and 276 cases in the prospective cohort) as a subgroup. The missing values of other variables were filled in with the KNN. Then, we constructed models with/without molecular classification (41/40 variables) to compare the models’ performances. The results were recorded in the **Supplementary Table 2**. The models with/without molecular classification obtained similar AUC values (0.902 and 0.871 in the internal validation cohort and 0.761 and 0.635 in the external validation cohort). The sensitivity of the models based on the same cohorts with or without molecular classification was still satisfactory (both 1 in the internal and external validation cohort). Also, the model with/without molecular classification had similar precision and overall accuracy in internal and external validation cohorts. The p-value of the Delong test indicated a significant difference between the two models in the external validation cohort (p=0.004). At the same time, there’s no significant difference in the internal validation cohort (p=0.470). Notably, although the performance of the model including molecular classification showed no significant difference in internal validation cohort, but it demonstrated a trend of higher AUC values in model including molecular classification in both two validation cohorts.

### 3.4 The construction of the simplified nomogram

**Figure 3:**
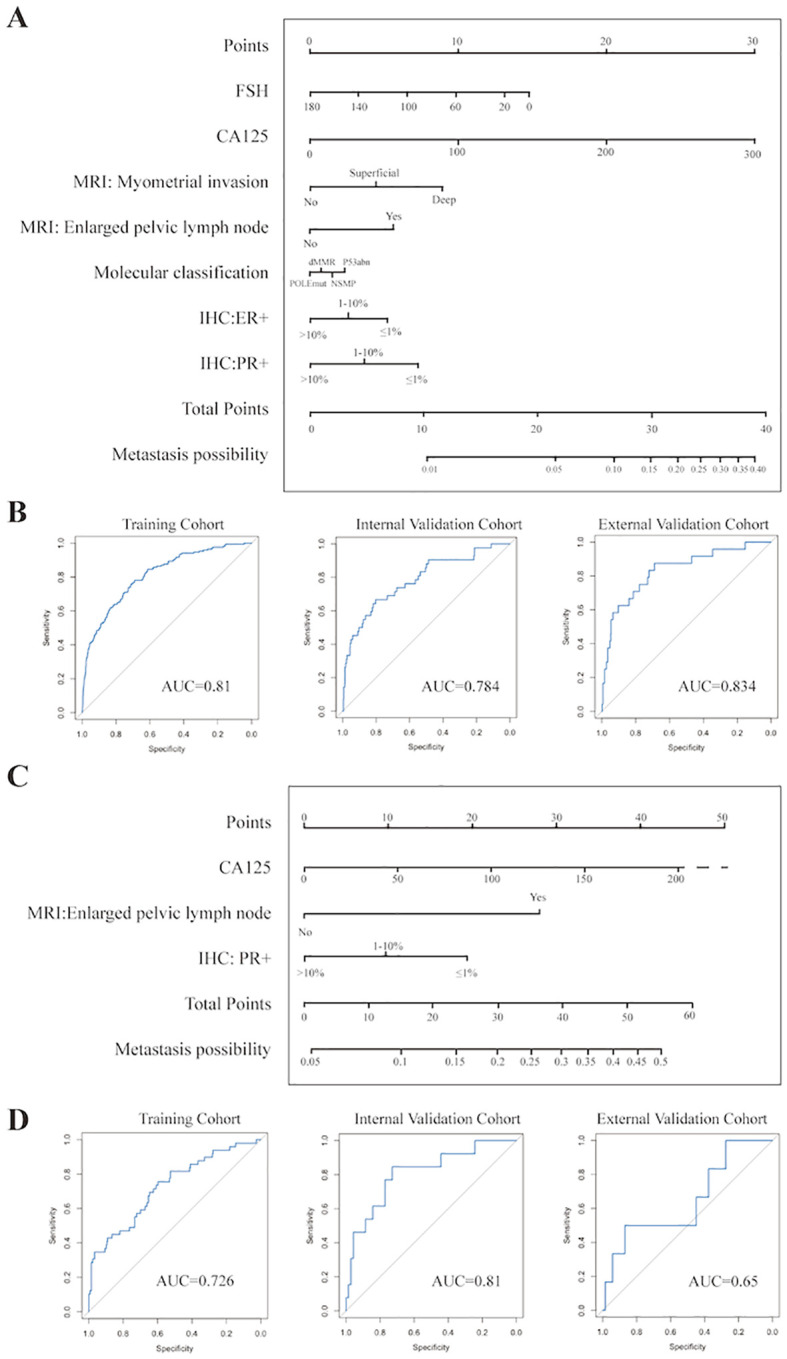
Establishment and Evaluation of Nomograms **(A)** Nomogram for prediction of lymph node metastasis in non-aggressive pathological subtypes. **(B)** ROC curves of nomogram of non-aggressive pathological subtypes in the training, internal validation, and prospective external validation cohorts. **(C)** Nomogram for prediction of lymph node metastasis in aggressive pathological subtypes. **(D)** ROC curves of nomogram of aggressive pathological subtypes in the training, internal validation, and prospective external validation cohorts.

Combining the results of Logistic regression and the Top 10 ranked variables (as shown in **Figures 2b–d**, details were recorded in **Supplementary Table 3**) by weights in FC Network, the simplified nomograms were designed to serve as the practical alternatives to the model in certain situations. The variables included in the nomograms model were determined by taking the intersection of those that remained statistically significant in both single factor and multi-factor logistic regression analyses and the top 10 weighted variables from the fully-connected network. Considering the different clinical prognoses associated with different histological subtypes and recognizing that the nomogram model involves a more simplified variable selection process compared to the FC Network, there is a potential risk of omitting critical features related to histological type. To enhance the predictive accuracy and applicability, we further stratified patients into non-aggressive types (including low-grade G1 and G2 endometrioid carcinoma) and aggressive types (including serous, high-grade endometrioid, clear cell, mixed and the other subtypes) (26), and constructed separate nomogram models for each group, as shown in **Figure 3**. **Figure 3A** illustrates the nomogram model for patients with non-aggressive histologic type. This model was constructed based on the top ten weighted variables from the FC network, combined with those that remained statistically significant in both single factor and multi-factor logistic regression analyses: FSH, CA125, MRI-indicated myometrial invasion, MRI-indicated enlarged pelvic lymph nodes, PR positivity, ER positivity, and molecular classification. The corresponding scores are detailed in the **Supplementary Tables 4–6**. The model yielded AUCs of 0.810, 0.784, and 0.834 in the training, internal validation, and external validation cohorts, respectively (**Figure 3B**). **Figure 3C** presents the nomogram model for patients with aggressive histologic type. The details of single factor and multi-factor analyses were in the **Supplementary Tables 7, 8**. The final variables selected were CA125, MRI-indicated enlarged pelvic lymph nodes, and PR positivity. The scores were recorded in **Supplementary Table 9**. The AUCs achieved in the training, internal validation, and external validation cohorts were 0.726, 0.810, and 0.650, respectively (**Figure 3D**). Despite the limited number of predictive variables in this subgroup, findings such as pelvic lymph node enlargement and low PR expression may serve as important red flags for increased metastatic risk, highlighting the need for vigilant clinical assessment. The simplified nomogram models for both non-aggressive and aggressive histological subtypes served as practical alternatives or complements to the fully-connected network model, providing a more user-friendly tool for clinical risk stratification.

## 4 Discussion

A predictive model for lymph node metastasis in early-stage endometrial cancer patients was developed using cohorts from two centers. The model exhibited high sensitivity—0.982 in internal validation and 0.900 in external validation—demonstrating superior performance in identifying patients with probable lymph node metastasis compared to other machine-learning methods. The addition of molecular classification may enhance the predictive power of the model. To facilitate clinical application, we also constructed the simplified nomograms based on different pathological subtypes by combining results from the top 10 risk factors ranked by their weights in the FC Network and risk factors correlated with lymph node metastasis in logistic regression model. The model can assist in decision-making before surgery for patients with early-stage endometrial cancer whose status of lymph nodes could not be evaluated by imaging or other preoperative examinations. To support clinical decision-making, our model stratifies patients based on preoperative risk, suggesting full lymphadenectomy for high-risk cases and sentinel lymph node biopsy for those at lower risk. For patients with a risk less than 1%, only total hysterectomy and no need for a sentinel lymph node biopsy. For those early-stage patients with a risk of greater than 1% and less than 5%, despite of total hysterectomy, sentinel lymph node biopsy could be considered. For those with a risk of 5-25%, sentinel lymph node biopsy is highly recommended besides total hysterectomy. Total hysterectomy with complete lymph node dissection can be considered in patients with a risk of greater than 25%.

The surgical strategy chosen for patients with early-stage endometrial cancer significantly impacts their prognosis (27). Performing lymph node dissection on all clinical early-stage patients without proper risk stratification may lead to overtreatment, as research has shown that sentinel lymph node biopsy is an acceptable alternative (28, 29). Conversely, failing to assess lymph nodes in patients who do have metastasis can increase the likelihood of postoperative recurrence. Therefore, accurately predicting the risk of lymph node metastasis in these patients is vital for improving their outcomes.

Several studies have addressed this problem. One study by KGOG used features from MRI, preoperative biopsy, and serum CA125 to establish criteria for risk categorization (30). These criteria obtained an excellent sensitivity of 0.849 in their study. Another study used variables mainly from pathological results and CA125 to construct a nomogram for risk-stratification and received a good AUC of 0.84 and 0.75 in high-grade and low-grade EC groups (31). This research was mainly based on the classic variables in endometrial cancer, and the models proposed in their studies performed well in prediction. However, this introduces a new challenge for surgeons: choosing between models. With patients undergoing more comprehensive preoperative examinations, more variables are available, and multiple models show good performance. As a result, researchers have become more aware of the need to compare predictive models and have observed fluctuating sensitivities of different models within the same cohorts (32).

Therefore, when constructing the model, we included as many classic variables as possible to utilize better the information that can be obtained before surgery to depict the unique evaluation for each patient precisely and included some newly proposed and widely recognized factors, such as molecular classification, for prediction. Different molecular and pathological subtypes of endometrial cancer carry distinct risk profiles; thus, we incorporated both as input variables in our model to enhance its generalizability. In clinical practice, patients with clear evidence of extrauterine spread—such as lymph node metastasis—on imaging are typically managed with lymph node dissection during surgery (33). Consequently, these patients were excluded from our study cohorts to focus on cases where lymph node status could not be determined preoperatively through imaging.

Due to the extended timeframe of the cohorts in this study and the fact that some examinations have only recently become widely available, certain data are missing. As previously mentioned, molecular classification has increasingly gained acceptance among clinicians in recent years and plays a crucial role in predicting the prognosis of endometrial cancer (34, 35). We included molecular classification as one of the variables to align with the future trend of risk stratification in endometrial cancer, aiming to enhance predictive efficiency. However, it is important to note that our implementation method may not be suitable for all situations, as new algorithms have emerged that have proven reliable and can reduce the number of required tests without affecting risk stratification (36, 37). We also compared the model’s sensitivity in subgroups with/without molecular classification. The addition of molecular classification showed higher AUC value in larger cohort and there was statistical difference between the two models in external validation cohort. But due to the limited number of cases, further research is needed to verify molecular classification’s role in predicting lymph node metastasis.

Recognizing the prognostic heterogeneity among histological subtypes and the more simplified feature selection in the nomogram compared to the FCN, we further stratified patients into non-aggressive and aggressive types and constructed subtype-specific nomograms accordingly. For non-aggressive subtypes, the final model incorporated FSH, CA125, MRI-indicated myometrial invasion, enlarged pelvic lymph nodes, ER/PR positivity, and molecular classification. Notably, in estrogen-dependent endometrial cancers, the predictive value of molecular classification—as well as ER and PR expression—proved to be non-negligible. In contrast, the aggressive subtype model retained only CA125, enlarged pelvic lymph nodes, and PR positivity as predictors. Due to the limited sample size in this subgroup, fewer variables were incorporated. Nevertheless, among these patients, ER expression appeared to be less predictive, while CA125 levels remained clinically relevant. More importantly, imaging-detected pelvic lymph node enlargement and reduced PR expression were frequently associated with lymph node metastasis. These findings suggest that, in aggressive subtypes, clinicians should pay particular attention to these two factors during preoperative evaluation. These variables also have similarities with the treatment guidelines and other studies, emphasizing the importance of imaging examinations in preoperative evaluation (38–40).

This study has the following highlights: firstly, the model integrates different types of variables, including widely used and newly upcoming variables, which would use as much as possible variables to evaluate each single patient, and the results showed the superiority to other conventional machine-learning methods. Secondly, the cohorts excluded patients with apparent extrauterine metastases, which corresponds to the dilemma of surgical strategy decision before the surgery as whether lymph node metastasis occurred or not and has a good guiding role for preoperative evaluation. Finally, for the convenience of application, we constructed a simplified nomogram, and its reliability has been verified. The limitations of this study mainly are: (1) Although imputing missing values is recognized, complete original data remains indispensable, which would definitely improve the performance of the model in real application, especially given the limited data available for molecular classification; (2) The number of cases in the external validation cohort is small; enrolling more cases would improve the accuracy of the results.

Overall, the model proposed in this study for preoperative prediction on the risk of lymph node metastasis in early-stage endometrial cancer patients can provide clinicians with preoperative reference to determine surgical approach or other adjuvant treatment options, considering the high sensitivity in screening occult lymph node metastasis patients. Also, the nomograms proposed in this study can be applied as an alternative to the Fully-connected Network model in certain situations.

## Data Availability

The raw data supporting the conclusions of this article will be made available by the authors, without undue reservation.
