# Supplementary material for: Fully-connected network-based prediction model for lymph node metastasis in clinical early-stage endometrial cancer: development and validation in two centers
Source: Front Oncol. 2025 Aug 25;15:1627662. doi: 10.3389/fonc.2025.1627662 (PMC12414781; doi:10.3389/fonc.2025.1627662)
Supplement: Supplementary file 1 [file Table1.docx]

Supplementary Table 1 Comparison of Different Missing Data Fill-in Methods

| Method | Sensitivity | Specificity | Precision | Overall  Accuracy | AUC |
| --- | --- | --- | --- | --- | --- |
| Mean | 0.982 | 0.067 | 0.074 | 0.131 | 0.749 |
| Median | 0.964 | 0.121 | 0.076 | 0.180 | 0.773 |
| Most Frequent | 0.964 | 0.060 | 0.072 | 0.124 | 0.704 |
| Constant | 0.982 | 0.126 | 0.078 | 0.186 | 0.703 |
| KNN | 0.982 | 0.074 | 0.074 | 0.138 | 0.746 |

**Supplementary Table 2 Comparison of Models With or Without Molecular Classification**

| **Subgroup** | **Cohort** | **Variables** | **Overall**  **Accuracy** | **Precision** | **Sensitivity** | **AUC** | **P Value** |
| --- | --- | --- | --- | --- | --- | --- | --- |
| Subgroup:  With complete molecular classification data  (n=616, 340 cases from retrospective cohort and 276 cases from prospective cohort) | Internal Validation  (n=68) | 41 Variables (with molecular classification) | 0.103 | 0.103 | 1 | 0.902 | 0.470 |
|  |  | 40 Variables (without molecular classification) | 0.103 | 0.103 | 1 | 0.871 |  |
|  | External Validation  (Prospective)  (n=276) | 41 Variables (with molecular classification) | 0.080 | 0.080 | 1 | 0.761 | 0.004 |
|  |  | 40 Variables (without molecular classification) | 0.083 | 0.080 | 1 | 0.635 |  |

Note: Other variables were filled in by using Median. Delong test was used.

Supplementary Table 3 Weights of Variables in Fully-connected Network

| Fig1 axis-x | Fig1 Weight | Fig2 axis-x | Fig2 Weight | Fig3 axis-x | Fig3 Weight |
| --- | --- | --- | --- | --- | --- |
| Age | 3.01E-05 | Age | 3.01E-05 | Age | 0.002091577 |
| Height | 0.004682561 | Height | 0.004682561 | Height | 8.56E-06 |
| Weight | 0.000167319 | Weight | 0.000167319 | Weight | 0.013139563 |
| BMI | 0.000376775 | BMI | 0.000376775 | BMI | 0.003968061 |
| preop_pathology-Endometroid | 0.008682759 | preop_pathology | 0.026567725 | preop_pathology | 0.000707552 |
| preop_pathology-Serous | 0.002764464 | Menopause | 0.005296478 | Menopause | 0.000906146 |
| preop_pathology-Mixed | 0.004147251 | Hypertension | 0.00256426 | Hypertension | 0.000476046 |
| preop_pathology-Clear cell | 0.000215124 | Diabetes | 0.005431003 | Diabetes | 0.000446791 |
| preop_pathology-High grade adenocarcinoma | 0.008505375 | FPG | 0.000677775 | FPG | 0.000506508 |
| preop_pathology-Other | 0.002252751 | HBA1C | 0.000756915 | HBA1C | 0.000350151 |
| Menopause-no | 0.003291021 | E2 | 8.39E-07 | E2 | 0.035448294 |
| Menopause-yes | 0.002005457 | P | 0.000159446 | P | 0.002880847 |
| Hypertension-no | 0.001623175 | T | 0.001085658 | T | 0.000415315 |
| Hypertension-yes | 0.000941085 | FSH | 2.28E-05 | FSH | 0.00999512 |
| Diabetes-no | 0.003230878 | LH | 3.17E-05 | LH | 0.002428052 |
| Diabetes-yes | 0.002200125 | SHBG | 1.35E-05 | SHBG | 0.004430585 |
| FPG | 0.000677775 | TG | 0.000133433 | TG | 0.00043467 |
| HBA1C | 0.000756915 | TC | 0.00012163 | TC | 0.000589993 |
| E2 | 8.39E-07 | HDL | 0.000202437 | HDL | 0.000225269 |
| P | 0.000159446 | LDL | 3.23E-05 | LDL | 0.000408386 |
| T | 0.001085658 | APOA | 0.00027348 | APOA | 0.000505396 |
| FSH | 2.28E-05 | APOB | 0.000145172 | APOB | 0.000104456 |
| LH | 3.17E-05 | ALP | 1.11E-05 | ALP | 0.00710351 |
| SHBG | 1.35E-05 | CA125 | 3.49E-05 | CA125 | 0.034602948 |
| TG | 0.000133433 | HE4 | 4.49E-06 | HE4 | 0.023821454 |
| TC | 0.00012163 | MRI_Tumor_size | 0.008155702 | MRI_Tumor_size | 0.001877079 |
| HDL | 0.000202437 | MRI_Myometrial_invasion | 0.015380752 | MRI_Myometrial_invasion | 0.001437057 |
| LDL | 3.23E-05 | MRI_Cervical_involvement | 0.003753745 | MRI_Cervical_involvement | 7.85E-05 |
| APOA | 0.00027348 | MRI_PVLN | 0.016119498 | MRI_PVLN | 0.000235203 |
| APOB | 0.000145172 | MRI_PALN | 0.019121783 | MRI_PALN | 1.32E-06 |
| ALP | 1.11E-05 | ultrasound_endometrium | 0.000119918 | ultrasound_endometrium | 0.003056305 |
| CA125 | 3.49E-05 | ultrasound_Tumor_size | 0.002353919 | ultrasound_Tumor_size | 0.000212229 |
| HE4 | 4.49E-06 | ultrasound_Myometrial_invasion | 0.004837539 | ultrasound_Myometrial_invasion | 0.00060404 |
| MRI_Tumor_size-＜2cm | 0.004904307 | CT_Posterior_peritoneum_LN | 0.001040821 | CT_Posterior_peritoneum_LN | 7.78E-05 |
| MRI_Tumor_size-≥2cm | 0.003251395 | MMR | 0.006261248 | MMR | 0.001157166 |
| MRI_Myometrial_invasion-No | 0.005290627 | P53 | 0.004350404 | P53 | 0.000520507 |
| MRI_Myometrial_invasion-Superficial | 0.001034332 | PTEN | 0.00107416 | PTEN | 8.19E-05 |
| MRI_Myometrial_invasion-Deep | 0.009055794 | Molecular_type | 0.011602151 | Molecular_type | 0.000993207 |
| MRI_Cervical_involvement-no | 0.003083383 | ER | 0.017166951 | ER | 0.001382763 |
| MRI_Cervical_involvement-yes | 0.000670362 | PR | 0.018722164 | PR | 0.002599812 |
| MRI_PVLN-No | 0.00835582 | Ki67 | 0.004210645 | Ki67 | 0.00018147 |
| MRI_PVLN-Enlarged | 0.007763678 |  |  |  |  |
| MRI_PALN-No | 0.012884993 |  |  |  |  |
| MRI_PALN-Enlarged | 0.00623679 |  |  |  |  |
| ultrasound_endometrium | 0.000119918 |  |  |  |  |
| ultrasound_Tumor_size:<2cm | 0.002132258 |  |  |  |  |
| ultrasound_Tumor_size:≥2cm | 0.000221661 |  |  |  |  |
| ultrasound_Myometrial_invasion-No | 0.001880284 |  |  |  |  |
| ultrasound_Myometrial_invasion-Unclear | 0.00070224 |  |  |  |  |
| ultrasound_Myometrial_invasion-Myometrial invasion | 0.002255015 |  |  |  |  |
| CT_Posterior_peritoneum_LN-No | 0.000518417 |  |  |  |  |
| CT_Posterior_peritoneum_LN-Enlarged | 0.000522403 |  |  |  |  |
| MMR-dMMR | 0.003108519 |  |  |  |  |
| MMR-pMMR | 0.003152729 |  |  |  |  |
| P53-Wild type | 0.002445839 |  |  |  |  |
| P53-Mutant | 0.001904565 |  |  |  |  |
| PTEN-no | 0.000528975 |  |  |  |  |
| PTEN-yes | 0.000545185 |  |  |  |  |
| Molecular_type-POLE | 0.000363824 |  |  |  |  |
| Molecular_type-MSI-H | 0.003002469 |  |  |  |  |
| Molecular_type-NSMP | 0.006143432 |  |  |  |  |
| Molecular_type-p53 | 0.002092425 |  |  |  |  |
| ER-≤1% | 0.010208695 |  |  |  |  |
| ER-1-10% | 0.001217885 |  |  |  |  |
| ER->10% | 0.005740371 |  |  |  |  |
| PR-≤1% | 0.010365207 |  |  |  |  |
| PR-1-10% | 0.000420082 |  |  |  |  |
| PR->10% | 0.007936874 |  |  |  |  |
| Ki67 | 0.004210645 |  |  |  |  |

Supplementary Table 4 P Values of Single-factor Logistic Regression in Training Cohort (non-aggressive)

| **Variable** | **P Value** | **Variable** | **P Value** |
| --- | --- | --- | --- |
| **General Information** |  | CA125 | <0.001 |
| Age | 0.051 | HE4 | 0.027 |
| Height | 0.748 | **Preoperative MRI** |  |
| Weight | 0.603 | Diameter of Tumor | <0.001 |
| BMI | 0.658 | Myometrial Invasion | <0.001 |
| Menopause | 0.804 | Cervical Involvement | <0.001 |
| Hypertension | 0.685 | Enlarged Pelvic Lymph Node | <0.001 |
| Diabetes | 0.145 | Enlarged Para-aortic Lymph Node | 0.005 |
| **Laboratory Test** |  | **Preoperative CT of Upper Abdomen** |  |
| FPG | 0.007 | Enlarged Retroperitoneal Lymph Node | 0.038 |
| HBA1C | 0.074 | **Preoperative Ultrasound** |  |
| E2 | 0.723 | Thickness of Endometrium | 0.005 |
| P | 0.564 | Diameter of Tumor | 0.022 |
| T | 0.238 | Lesion-myometrial Interface | 0.031 |
| FSH | 0.031 | **Immunohistochemistry** |  |
| LH | 0.103 | MMR | 0.033 |
| SHBG | 0.450 | P53 | 0.873 |
| TG | 0.885 | PTEN | 0.397 |
| TC | 0.829 | ER | <0.001 |
| HDL | 0.389 | PR | <0.001 |
| LDL | 0.115 | Ki67 | 0.005 |
| APOA | 0.147 | **Molecular Classification** | 0.009 |
| APOB | 0.792 |  |  |
| ALP | 0.069 |  |  |

Note: P value showed the statistical analysis between training cohort and validation cohort. For variables conforming to normal distribution, t-test was used. For continuous variables that did not conform to normal distribution, Mann-Whitney test was used. And for categorical variables, the chi-square test was used. Statistical significance was set at P <0 .05 (2-tailed)

Supplementary Table 5 P Values of Multi-factor Logistic Regression in Training Cohort (non-aggressive)

| **Variable** | **P** | **Variable** | **P** |
| --- | --- | --- | --- |
| **Laboratory Test** |  | Ultrasound: Lesion-myometrial Interface | 0.826 |
| FPG | 0.048 | Clear | Ref |
| FSH | 0.005 | Unclear | 0.554 |
| CA125 | <0.001 | Myometrial Invasion | 0.942 |
| HE4 | 0.363 | **Immunohistochemistry** |  |
| **Preoperative MRI** |  | ER | 0.009 |
| MRI: Size of Tumor | 0.360 | ≤1% | 0.004 |
| MRI: Myometrial Invasion | <0.001 | 1-10% | 0.093 |
| No Myometrial Invasion | Ref | >10% | Ref |
| Shallow Myometrial Invasion | 0.001 | PR | 0.007 |
| Deep Myometrial Invasion | <0.001 | ≤1% | 0.090 |
| MRI: Cervical Involvement | 0.802 | 1-10% | 0.002 |
| MRI: Enlarged Pelvic Lymph Node | 0.025 | >10% | Ref |
| MRI: Enlarged Para-aortic Lymph Node | 0.150 | MMR | 0.238 |
| **Preoperative CT of Upper Abdomen** |  | Ki67 | 0.166 |
| Enlarged Retroperitoneal Lymph Node | 0.668 | **Molecular Classification** | 0.035 |
| **Preoperative Ultrasound** |  | POLE*mut* | Ref |
| Ultrasound: Thickness of Endometrium | 0.176 | dMMR | 0.574 |
| Ultrasound: Size of Tumor | 0.787 | NSMP | 0.272 |
|  |  | P53abn | 0.792 |

Supplementary Table 6 Points of Each Variables in the Nomogram (non-aggressive)

| **Variable** | **Scores** |
| --- | --- |
| **FSH** | 14.78-Value*0.082 |
| **CA125** | Value*0.1 |
| **MRI：Myometrial Invasion** |  |
| No Myometrial Invasion | 0 |
| Superficial Myometrial Invasion | 4.46 |
| Deep Myometrial Invasion | 8.93 |
| **MRI: Enlarged Pelvic Lymph Node** |  |
| No | 0 |
| Yes | 5.6 |
| **IHC: PR+** |  |
| >10% | 0 |
| 1-10% | 2.60 |
| ≤1% | 5.21 |
| **IHC: ER+** |  |
| >10% | 0 |
| 1-10% | 3.65 |
| ≤1% | 7.30 |
| **Molecular Classification** |  |
| POLE*mut* | 0 |
| dMMR | 0.76 |
| NSMP | 1.52 |
| P53abn | 2.28 |
| **Risk of Lymph Node Metastasis** |  |
| <1% | <10.2 |
| 1-5% | 10.2-21.6 |
| 5-25% | 21.6-34.3 |
| ≥25% | ≥34.3 |

Supplementary Table 7 P Values of Single-factor Logistic Regression in Training Cohort (aggressive)

| **Variable** | **P Value** | **Variable** | **P Value** |
| --- | --- | --- | --- |
| **General Information** |  | CA125 | <0.001 |
| Age | 0.102 | HE4 | <0.001 |
| Height | 0.738 | **Preoperative MRI** |  |
| Weight | 0.526 | Diameter of Tumor | 0.001 |
| BMI | 0.384 | Myometrial Invasion | <0.001 |
| Menopause | 0.087 | Cervical Involvement | 0.062 |
| Hypertension | 0.639 | Enlarged Pelvic Lymph Node | <0.001 |
| Diabetes | 0.141 | Enlarged Para-aortic Lymph Node | / |
| **Laboratory Test** |  | **Preoperative CT of Upper Abdomen** |  |
| FPG | 0.194 | Enlarged Retroperitoneal Lymph Node | 0.047 |
| HBA1C | 0.809 | **Preoperative Ultrasound** |  |
| E2 | 0.650 | Thickness of Endometrium | 0.969 |
| P | 0.891 | Diameter of Tumor | 0.639 |
| T | 0.279 | Lesion-myometrial Interface | 0.347 |
| FSH | 0.550 | **Preoperative Pathological Type** | 0.469 |
| LH | 0.945 | **Immunohistochemistry** |  |
| SHBG | 0.658 | MMR | 0.841 |
| TG | 0.062 | P53 | 0.983 |
| TC | 0.909 | PTEN | 0.820 |
| HDL | 0.225 | ER | 0.270 |
| LDL | 0.440 | PR | 0.008 |
| APOA | 0.403 | Ki67 | 0.184 |
| APOB | 0.639 | **Molecular Classification** | 0.408 |
| ALP | 0.507 |  |  |

Note: P value showed the statistical analysis between training cohort and validation cohort. For variables conforming to normal distribution, t-test was used. For continuous variables that did not conform to normal distribution, Mann-Whitney test was used. And for categorical variables, the chi-square test was used. Statistical significance was set at P <0 .05 (2-tailed)

Supplementary Table 8 P Values of Multi-factor Logistic Regression in Training Cohort (aggressive)

| **Variable** | **P** | **Variable** | **P** |
| --- | --- | --- | --- |
| **Laboratory Test** |  | **Preoperative CT of Upper Abdomen** |  |
| CA125 | 0.008 | CT: Enlarged Retroperitoneal Lymph Node | 0.823 |
| HE4 | 0.078 | **Immunohistochemistry** |  |
| **Preoperative MRI** |  | PR | 0.041 |
| MRI: Size of Tumor | 0.134 | ≤1% | 0.018 |
| MRI: Myometrial Invasion | 0.120 | 1-10% | 0.400 |
| No Myometrial Invasion | Ref | >10% | Ref |
| Shallow Myometrial Invasion | 0.849 |  |  |
| Deep Myometrial Invasion | 0.079 |  |  |
| MRI: Enlarged Pelvic Lymph Node | 0.027 |  |  |

Supplementary Table 9 Points of Each Variables in the Nomogram (aggressive)

| **Variable** | **Scores** |
| --- | --- |
| **CA125** | Value*0.222 |
| **MRI: Enlarged Pelvic Lymph Node** |  |
| No | 0 |
| Yes | 28 |
| **IHC: PR+** |  |
| >10% | 0 |
| 1-10% | 9.65 |
| ≤1% | 19.30 |
| **Risk of Lymph Node Metastasis** |  |
| 1% | / |
| <5% | <0.679 |
| 5-25% | 0.679-34.442 |
| ≥25% | ≥34.442 |
