# Supplementary figures and images for: Fully-connected network-based prediction model for lymph node metastasis in clinical early-stage endometrial cancer: development and validation in two centers
Source: Front Oncol. 2025 Aug 25;15:1627662. doi: 10.3389/fonc.2025.1627662 (PMC12414781; doi:10.3389/fonc.2025.1627662)

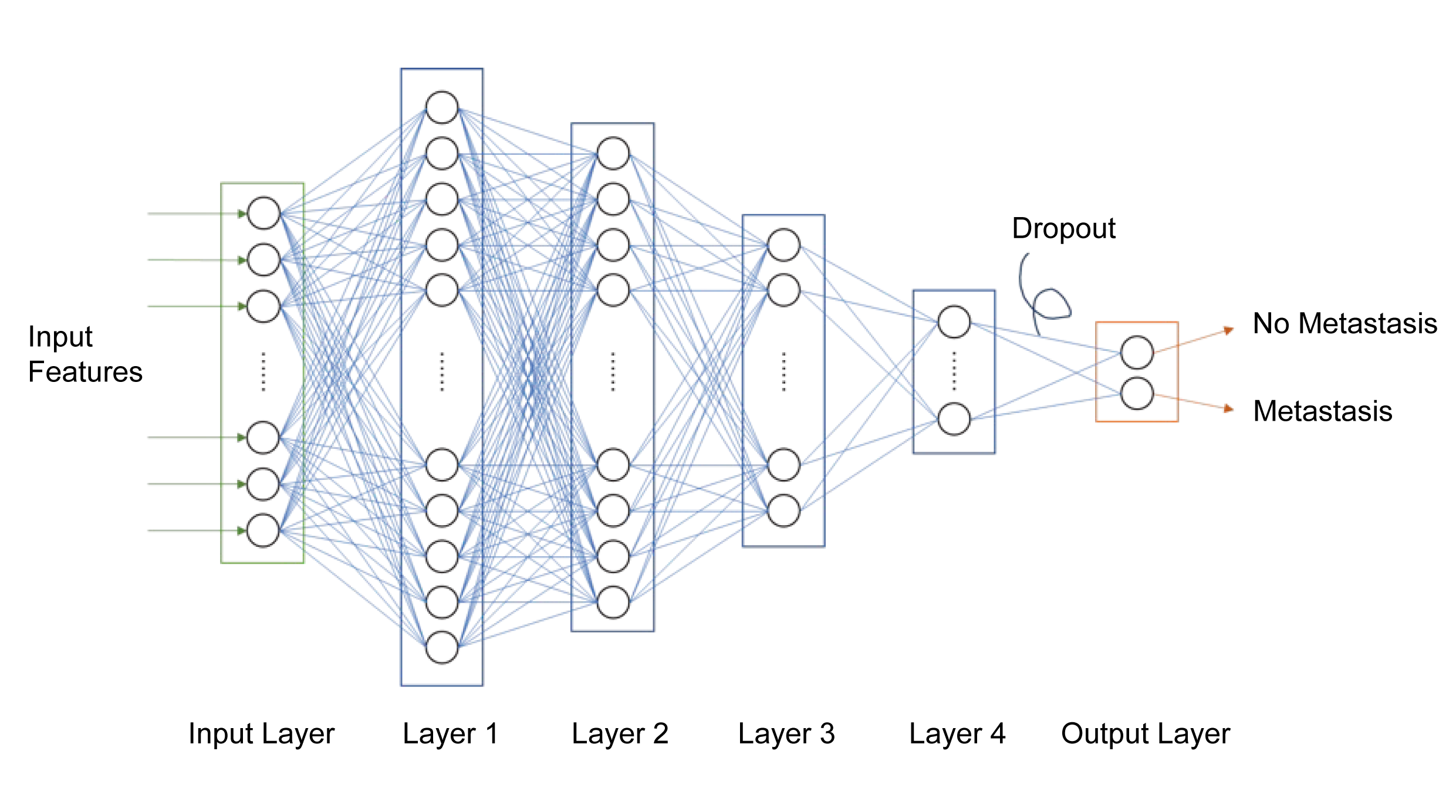

Supplement: Supplementary file 2 [file Image1.tif]

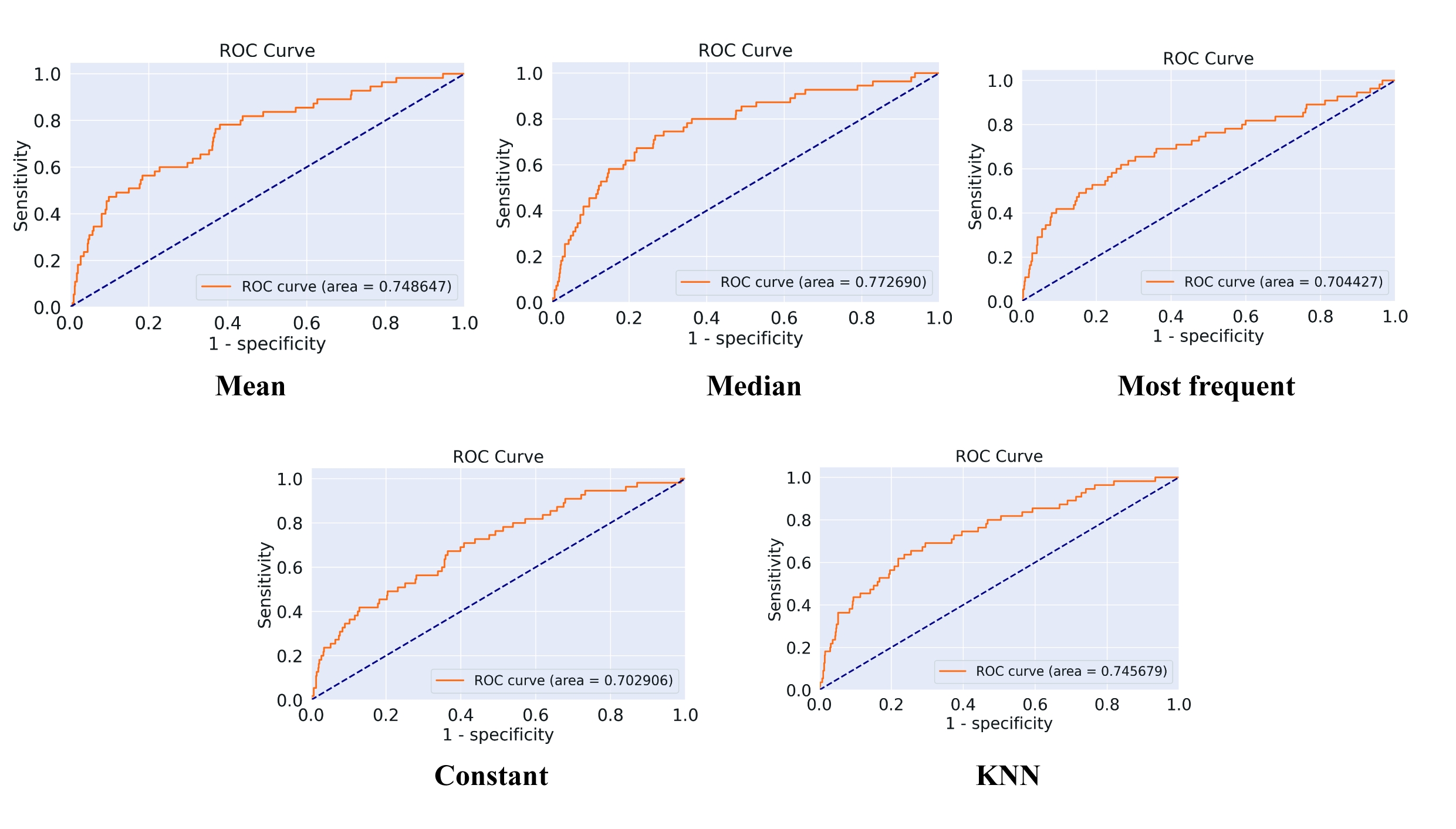

Supplement: Supplementary file 3 [file Image2.tif]
